# Takayasu Arteritis Coexisting with Cutaneous Leishmaniasis

**DOI:** 10.3390/jcm12051819

**Published:** 2023-02-24

**Authors:** Mutong Zhao, Ying Liu, Zhihai Hu, Juan Sun, Zhou Yang, Li Wei, Zigang Xu, Lin Ma

**Affiliations:** 1 Department of Dermatology, Beijing Children’s Hospital, Capital Medical University, National Center for Children’s Health, Beijing 100045, China; 2 Department of Medical Imaging, Beijing Friendship Hospital, Capital Medical University, Beijing 100050, China

**Keywords:** leishmaniasis, antimony, Takayasu arteritis, inflammation, infection

## Abstract

Takayasu arteritis (TA) is a rare large-vessel vasculitis that can result in significant morbidity and mortality. The coexistence of TA with leishmaniasis infection has not been reported previously. Case description: An 8-year-old girl presented with recurrent skin nodules that heal spontaneously for four years. Her skin biopsy revealed granulomatous inflammation with Leishmania amastigotes identified in the histocyte cytoplasm and the extracellular space. The diagnosis of cutaneous leishmaniasis was made and intralesional sodium antimony gluconate was started. One month later, she experienced dry coughs and fever. The CT angiography of the carotid arteries showed dilation in the right common carotid artery and thickening of artery walls with elevated acute phase reactants. The diagnosis of Takayasu arteritis (TA) was made. Reviewing her chest CT before treatment, a soft-tissue density mass was identified in the right carotid artery region, suggesting a pre-existing aneurysm. The patient was treated with surgical resection of the aneurysm with systemic corticosteroids and immunosuppressants. Her skin nodules resolved with scars after the second cycle of antimony while a new aneurysm arose due to a lack of control of TA. Conclusions: This case highlights that benign as the natural course is for cutaneous leishmaniasis, fatal comorbidities can occur as a consequence of chronic inflammation, and can be aggravated by the treatment.

## 1. Introduction

Leishmaniases are a group of diseases caused by protozoan parasites from more than 20 Leishmania species. It was estimated that 700,000 to 1 million new cases occur annually worldwide [1]. Leishmania parasites are transmitted through the bites of infected female phlebotomine sandflies during a blood meal, allowing the transmission of promastigotes which reside in the midgut of the sandfly vector. Reservoirs include humans, dogs, leopards, hyenas, rodents, bats, and baboons. There are three forms of leishmaniases—the visceral, the cutaneous, and the mucocutaneous, with the cutaneous variant being the benign form. Cutaneous leishmaniasis is a neglected tropical disease that affects people worldwide. Because of the enhanced opportunity for exposure and possibility of not having a fully developed immune system, children may be more susceptible to infection than adults.

Takayasu arteritis (TA) is a form of granulomatous large-vessel arteritis with an ethnic predisposition in patients of Asian and Indian descent. TA significantly increases morbidity and mortality [2], with a 5-year mortality of up to 1.9%. While the pathogenesis of TA remains to be elucidated, infections of tuberculosis, chlamydia pneumoniae, mycoplasma, and Mycobacterium were suggested to be associated with an increased risk for TA [3].

We herein report a case of Takayasu arteritis coexisting with leishmaniasis infection, with disease relapse during antimony treatment. This case highlights that benign as the disease course is for cutaneous leishmaniasis, it is important to be alerted of fatal inflammatory comorbidities as inflammation always chaperones infection.

## 2. Case Description

**Figure 1:**
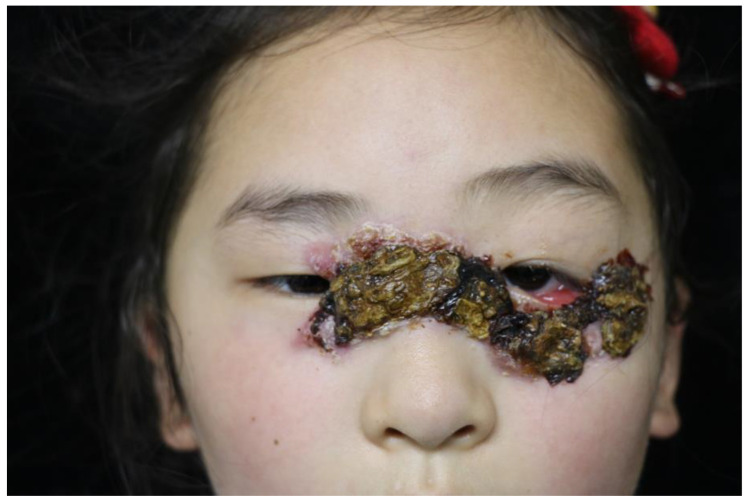
Disfiguring painless erythematous papules and nodules with overlying hyperkeratotic scales on the face of the patient.

**Figure 2:**
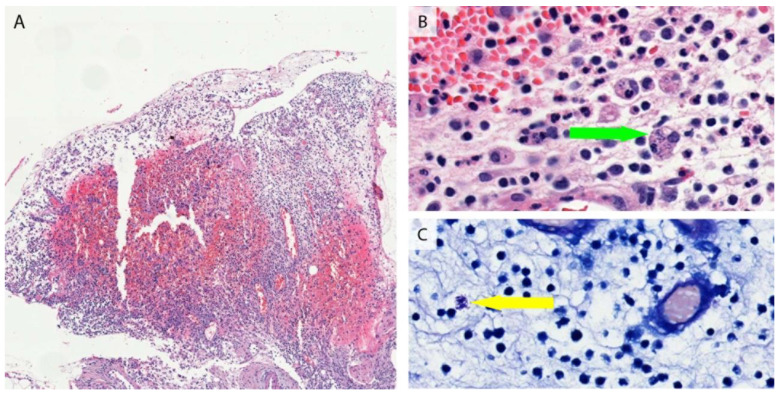
(**A**) Low-magnification HE staining showing a mixed infiltration of lymphocytes and histiocytes. (**B**) HE staining showing Leishmania amastigotes (arrow, ×400 magnification). (**C**) Giemsa staining showing Leishmania amastigotes (arrow, ×400 magnification).

An 8-year-old child presented with recurrent skin nodules that heal spontaneously with atrophic scars for four years (Figure 1). She was not responsive to topical steroids, nor to systemic treatments of cephalosporins and itraconazole. A short residence on a cargo ship that imports timber from West Africa (The Republic of Cameroon), which has been identified as an endemic region for leishmaniasis, was reported. No prior history of fever or weight loss was reported. Physical examination was notable for disfiguring painless erythematous papules and nodules with overlying hyperkeratotic scales (Figure 1). Vital signs were normal. Her skin biopsy revealed granulomatous inflammation with Leishmania amastigotes identified in the histocyte cytoplasm and the extracellular space (Figure 2A,B). Bone marrow aspiration was unremarkable and no evidence of anemia, hepatomegaly, or splenomegaly was present, which ruled out visceral leishmaniasis. The diagnosis of cutaneous leishmaniasis was made and intralesional sodium antimony gluconate (20 mg/kg daily for six consecutive days) was started with rapid improvement of the skin lesions.

**Figure 3:**
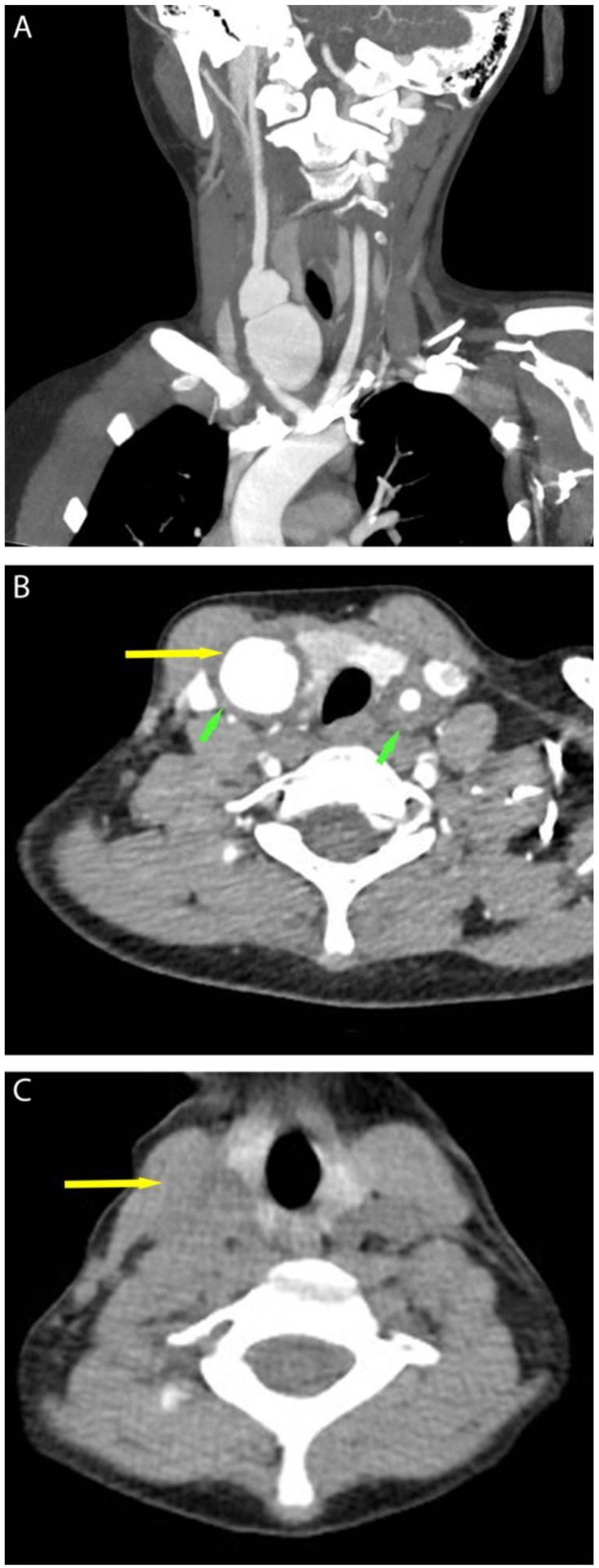
(**A**) Coronal plane of CT angiography showing dilation of the carotid arteries. (**B**) Transverse plane of CT angiography showing dilation (yellow arrow) and wall thickening (green arrows) of carotid arteries. (**C**) Chest CT before antimony treatment showing a pre-existing soft-tissue mass at the right common carotid artery region (arrow).

One month later, she experienced dry coughs, tachypnea, and recurrent fever. The vital signs were T 99.1 to 101.5 F, HR 123–138 bpm, BP 113–135/64–86, RR 17–26, and SpO_2_ 100% on room air. Chest CT revealed a soft-tissue mass in the neck next to the thyroid with color Doppler ultrasonography showing aneurysm formation with swirling blood flow. The intima media thickness (IMT) was measured to be: left carotid artery 0.28 cm, right carotid artery 0.16 cm. A CT angiography was requested, which confirmed the diagnosis of an aneurysm with a dilation of the right common carotid artery (Figure 3A). Wall thickening of the carotid arteries was noticed. ESR (80–140 mm/h, normal range: 0–20 mm/h) and CRP (11–190 mg/L, normal range: <8 mg/L) were significantly elevated. Based on the 2008 European League Against Rheumatism (EULAR) criteria for childhood TA [4], the diagnosis of Takayasu arteritis (TA) was made. Reviewing her chest CT before antimony administration, a soft-tissue density mass was identified in the right carotid artery region, suggesting a pre-existing aneurysm (Figure 3B); however, due to a lack of symptoms and low awareness of inflammatory comorbidities that may accompany chronic infectious diseases, this was overlooked. Other causes for aneurysms, including rheumatic disorders, hereditary connective tissue disorders (Marfan syndrome, Ehlers–Danlos syndromes, et al.), and other chronic infections associated with TA (syphilis, tuberculosis) were considered. Laboratory tests yielded the following: normal anti-nuclear antibodies, extractable nuclear antigens, complements, and antineutrophil cytoplasmic antibodies. Infectious screening tests for tuberculosis (tuberculin skin test and T-SPOT.TB) and syphilis (anti-TP-specific antibody) were negative. Genetic testing with Whole-Exome Sequencing (WES) was unremarkable.

**Figure 4:**
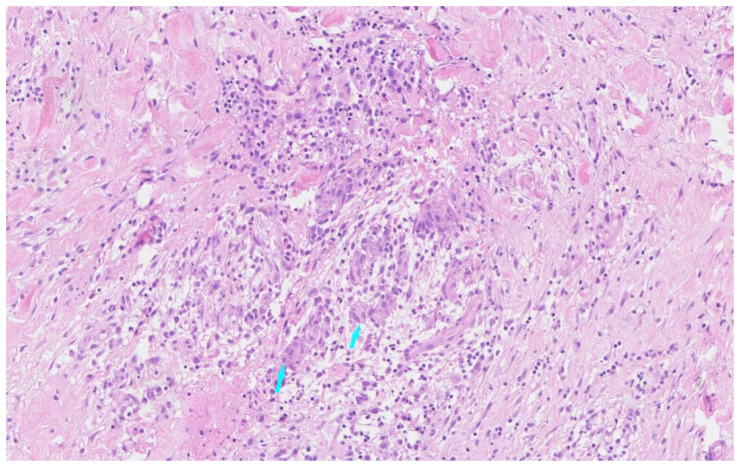
Histopathology of the aneurysm showing histiocytic and lymphocytic infiltration with multinucleate giant cells (blue arrows), ×200 magnification.

**Figure 5:**
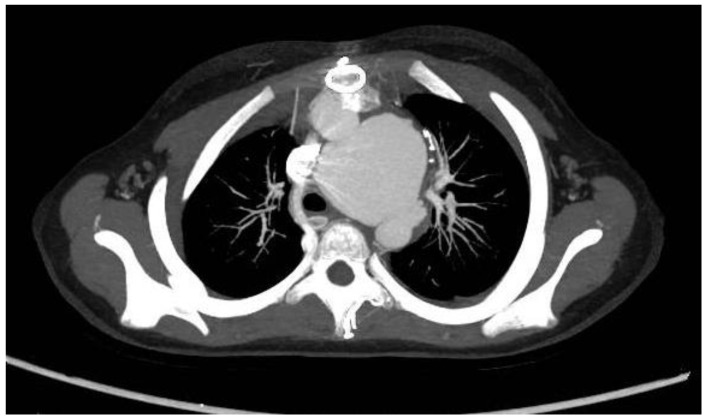
CT angiography of showing pseudoaneurysm of the aortic arch.

Considering that chronic infection might contribute to TA, and the close temporal association between antimony administration and the onset of TA relapse, the second cycle of antimony was postponed. A multidisciplinary consultation with rheumatologists, dermatologists, intensivists, and tropical disease specialists was held and the patient was subsequently treated with surgical resection of the aneurysm. Histopathology of the aneurysm showed histiocytic and lymphocytic infiltration (Figure 4) which further confirmed the diagnosis of TA. A pulsed methylprednisolone course of 20 mg/kg for 3 days, followed by 2 mg/kg daily, was started with mycophenolate mofetil added concurrently at a dose of 250 mg 3 times daily. Inflammatory markers were normalized within two months. The second cycle of antimony treatment was administered while methylprednisolone was gradually tapered off. Unfortunately, at the six-month follow-up check, while clinically stable, worsening of disease was evident with CT angiography notable for a progressively increasing wall thickening of the left aortic artery when methylprednisolone was tapered to 16 mg daily. A total of 200 mg of infliximab and 7.5 mg of methotrexate weekly were added on. Subsequently, the patient received 200 mg of infliximab at weeks 0, 2, 6, and 8 with weekly methotrexate while gradually tapering off methylprednisolone. ESR and CRP were normal. At the one-year follow-up, her skin nodules completely resolved with scars and no TA-related symptoms were present. The patient refused to undergo further surveillance of inflammatory markers due to COVID lockdowns and has declined further TA treatment ever since. At the writing of this case report, the 18-month follow-up was completed with CT angiography notable for an aneurysm of the aortic arch (Figure 5). ESR (87 mm/h) and CRP (>180 mg/L) were significantly elevated. The patient is currently under evaluation and weighing her treatment options.

## 3. Discussion

Our case described an 8-year-old leishmaniasis patient with a coexisting TA relapse during antimony treatment. Chronic infections are associated with a significantly higher risk of TA [5]. Our case was the first TA case known to coexist with leishmaniasis infection. Persistent infection with pathogens and chronic antigenic stimulation can activate vascular DCs, leading to large-vessel arteritis. However, prior to antimony treatment, this arteritis appeared to be in remission with no symptoms and only mildly elevated acute phase reactants (CRP of 10 mg/L, normal range <8 mg/L; ESR of 12 mm/h, normal range 0–20 mm/h). Upon treatment with antimony, metabolic perturbations of the parasite, i.e., long-chain fatty acid β-oxidation contribute to enhanced drug and ROS-mediated parasite killing [6]. Meanwhile, activation of the host immune system, including activation of macrophages, dendritic cells, T cells, and upregulation of proinflammatory cytokines contribute to clearance of infection [7]. Both ROS metabolites and the activated immune system may then in turn mediate vascular inflammation, leading to the onset or relapse of large-vessel arteritis [5,8]. The relapse of TA in the current patient was manifested by the increased size of the pre-existing aneurism, significantly elevated acute-phase reactants, and the recent onset of clinical symptoms. Treatment-associated inflammation exacerbation is best known in syphilis infection as the Jarisch–Herxheimer reaction. Cases have been reported on the presence of arteritis following syphilis treatment [9,10]. However, due to the limited evidence, we were not able to conclude on a causal relationship. Nonetheless, it is important to be alerted to the coexistence of fatal inflammatory comorbidities and infection, as inflammation always chaperones infection. Close monitoring should be implemented before and during treatment.

Treatment of TA during ongoing infection can be challenging. While clearance of leishmaniasis infection depends on the pro-inflammatory M1 macrophage polarization, mediators including cytokines such as TNF-α, IL-1β, IL-6, IL-12, and NO that kill the parasites also potently mediate tissue injury, culminating in possible aggravation of TA [11]. Meanwhile, inhibition of pro-inflammatory cytokines may facilitate parasite survival [11]. While TNF-alpha inhibition can be used as second-line treatment in case of relapsing disease [12], using TNF-alpha inhibition in the current patient was debated among the multidisciplinary team as a growing body of evidence suggested that greater risk for leishmaniasis can be associated with TNF-α inhibition [13]. Interestingly, this increase varies markedly between the different inhibitors currently in use with antibodies (e.g., infliximab and adalimumab) associated with approximately eight-fold odds of opportunistic leishmaniasis as opposed to etanercept [14]. This might be explained by the differences in the mode of action of different compounds. The anti-TNF antibodies bind to both the soluble and the transmembrane form of TNF, whereas etanercept targets mainly soluble TNF and interacts with the transmembrane form with reduced avidity. This can be beneficial for anti-leishmanial immunity, as transmembrane TNF but not soluble TNF is essential to control leishmaniasis infection [14]. In addition, the complement or antibody-dependent cellular cytotoxicity mediated by antibodies can induce apoptosis of pathogen-containing macrophages, thus leading to reactivation of latent infection [14]. While etanercept might be preferable in TA with leishmaniasis infection as a comorbidity, our patient chose to use infliximab due to the lower frequency of drug administration and traveling expenses. Considering the satisfactory efficacy of the first antimony treatment cycle, infliximab was added on and TA was controlled with no sign of leishmaniasis reactivation.

The long-term prognosis of TA can vary significantly. Ishikawa used a prognostic stratification system based on the presence of complications and the clinical course. In a group of patients who had major complications together with a progressive course, the 15-year survival rate was only 43% [15]. In contrast, for patients with no major complications, the 15-year survival rate was 96.4%. Similarly, in a more recent study by Mirouse, progressive disease course at diagnosis and complications of thoracic aorta involvement and retinopathy were independently associated with death and complication-free survival in the multivariate analysis [2]. With a progressive disease course complicated by the presence of an aortic arch aneurysm, the prognosis of the current patient is bleak.

## 4. Conclusions

TA and leishmaniasis are both rare diseases. Our case was the first TA case known to coexist with leishmaniasis infection. Cutaneous leishmaniasis, which is regarded as benign, can have fatal comorbidities due to the chaperoning inflammation, which can possibly be worsened by antileishmanial treatments. Close monitoring should be implemented for leishmaniasis patients at baseline and during treatment.

## Data Availability

Not applicable.
